# Understanding and Exploiting Transpiration Response to Vapor Pressure Deficit for Water Limited Environments

**DOI:** 10.3389/fpls.2022.893994

**Published:** 2022-05-10

**Authors:** Katrina J. Broughton, Warren C. Conaty

**Affiliations:** Agriculture and Food, CSIRO, Narrabri, NSW, Australia

**Keywords:** abiotic stress, crop/stress physiology, dryland, limited rate transpiration, rainfed, vapor pressure deficit

## Abstract

More frequent droughts and an increased pressure on water resources, combined with social licence to operate, will inevitably decrease water resources available for fully irrigated cotton production. Therefore, the long-term future of the cotton industry will require more drought tolerant varieties that can perform well when grown in rainfed cropping regions often exposed to intermittent drought. A trait that limits transpiration (TR_Lim_) under an increased vapour pressure deficit (VPD) may increase crop yield in drier atmospheric conditions and potentially conserve soil water to support crop growth later in the growing season. However, this trait has not been tested or identified in cotton production systems. This study tested the hypotheses that (1) genetic variability to the TR_Lim_ VPD trait exists amongst 10 genotypes in the Australian cotton breeding programme; (2) genotypes with a TR_Lim_ VPD trait use less water in high VPD environments and (3) variation in yield responses of cotton genotypes is linked with the VPD environment and water availability during the peak flowering period. This study combined glasshouse and field experiments to assess plant transpiration and crop yield responses of predominantly locally bred cotton genotypes to a range of atmospheric VPD under Australian climatic conditions. Results indicated that genetic variation to the limiting transpiration VPD trait exists within cotton genotypes in the Australian breeding programme, with five genotypes identified as expressing the TR_Lim_ VPD trait. A modelling study suggests that this trait may not necessarily result in overall reduced plant water use due to greater transpiration rates at lower VPD environments negating the water conservation in high VPD environments. However, our study showed that the yield response of cotton genotypes is linked with both VPD environment and water availability during the peak flowering period. Yield performance of the TR_Lim_ genotype was improved at some high VPD environments but is unlikely to out-perform a genotype with a lower yield potential. Improved understanding of integrated plant- and crop-level genotypic responses to the VPD environments will enhance germplasm development to benefit cotton production in both rainfed and semi-irrigated cotton systems, thereby meeting the agricultural challenges of the twenty-first Century.

## Introduction

Water deficits are a significant limitation to cotton production, where historically greater cotton yields have reflected access to irrigation water and in-season rainfall (Conaty et al., 2018). Recent prolonged droughts have placed increasing pressure on water resources for irrigated agriculture. This pressure, combined with social licence to operate, will presumably only decrease irrigation water available for cotton production and is similarly occurring across global cotton production regions (Hearn and Fitt, 1992; Hearn, 1994). Therefore, the long-term future of the cotton industry and its potential expansion into additional rainfed summer cropping regions will require the identification of drought tolerant varieties (Conaty et al., 2018). These varieties will be beneficial to cotton production in rainfed systems and could also impact production in limited-irrigation production environments.

Plants respond to changes in water availability in both their aerial and soil environments. The driving force of transpiration rate is the gradient in vapour pressure between the dry atmosphere and the wet interior of leaves, commonly referred to as the vapour pressure deficit (VPD). VPD is defined as the difference between the amount of moisture in the air at a given temperature and the amount of moisture the air can hold at the same temperature when it is saturated; thus, combining the effects of temperature and relative humidity. A high VPD indicates a hotter and drier environment, whilst a low VPD results from a cooler and more humid environment. Numerous studies have demonstrated that the stomata of cotton are highly responsive to changes in VPD (Yong et al., 1997; Devi and Reddy, 2018; Shekoofa et al., 2021), and VPD has been identified as a critical factor influencing transpiration and stomatal conductance in crops including cotton (Broughton et al., 2021) and maize (Yang et al., 2012).

Several adaptive strategies have evolved in plants to cope with drought stress. One of the strategies is to limit transpiration rate under high VPD environments (TR_Lim_ VPD), where plants may partially close their stomata to conserve soil water. The limitation of transpiration is one of the first responses observed in a plant under limited soil water conditions (drought) or high VPD conditions, before turgor loss or cavitation restricting water flow through the xylem, which is observed after prolonged or severe drought. Whilst limiting transpiration in these situations reduces photosynthetic performance, it is an adaptive strategy that helping to avoid lethal desiccation. Water is conserved for use when conditions are more favourable. This results in a more conservative crop growth rate, where soil water is not rapidly consumed, and thus if conservation of stored soil moisture occurs early in the growing season, there may be more water available later in the season to sustain plant physiological activity under dry conditions (Shekoofa et al., 2015). In addition, water conserved at high VPD conditions can also be used when environmental conditions ensure more efficient carbon assimilation with respect to crop water use. However, it is important to acknowledge that limited transpiration will also result in a decrease in photosynthetic rate, reducing yields compared with fully irrigated systems. Likewise, if no late-season water deficit develops, there would be no benefit from the conserved soil water, and a decrease in yield would be expected.

Genotypic variability has been observed for the TR_Lim_ VPD trait in response to high VPD in several crop species, including maize (Yang et al., 2012; Shekoofa et al., 2016b), sorghum (Gholipoor et al., 2010), soybean (Fletcher et al., 2007), peanut (Shekoofa et al., 2014) and pearl millet (Kholová et al., 2016). More recently, the trait has also been reported in cotton (Devi and Reddy, 2018). However, there have been limited studies examining variation of transpiration responses to VPD amongst Australian cotton genotypes and environments. Australia’s modern irrigated cotton industry developed in the 1960s in northern NSW and southern Queensland. The expansion of the industry was initially based on varieties from the United States; however, domestic breeding efforts led to the development of varieties more suited to the Australian environment (Constable et al., 2001; Liu et al., 2013). Presently, the Australian cotton industry extends from Central Queensland to Southern NSW, from subtropical to Mediterranean environments, so cotton is grown across locations of varied atmospheric demands. Recently, the industry has also started to expand into the wet-dry tropics of Northern Australia where broad acre cropping is being developed. Therefore, it is important to assess cotton germplasm for limiting transpiration traits in the context of the various Australian and global breeding target environments as well as in the context of projected future environments.

In this study, it was hypothesised that genetic variability to the TR_Lim_ VPD trait in response to atmospheric VPD exists within cotton germplasm. The aim of this study was (1) to identify the presence of the trait in cotton germplasm, as well as the degree that transpiration is limited in response to VPD and (2) to understand the implications of these transpiration responses for water use and yield of cotton grown in different VPD environments. We tested the hypotheses that (1) genetic variability to the TR_Lim_ VPD trait exists amongst 10 genotypes in the Australian cotton breeding programme, (2) genotypes with a TR_Lim_ VPD trait use less water in high VPD environments and (3) variation in yield responses of cotton genotypes is linked with the VPD environment and water availability during the peak flowering period. Our study integrated glasshouse experiments to measure plant-level transpiration of 10 genotypes in different VPD environments, modelling to estimate crop water-use of these genotypes in environmental conditions in the field in Narrabri, Australia, and tests against available yield data from multiple years and locations of two cotton genotypes with contrasting transpiration traits (TR_Linear_ and TR_Lim_). This study is important because the development of cultivars that can remain productive despite periods of water stress will be integral for cotton production in future and water limited environments. The integration of glasshouse and field studies, assessing genetic variability of cotton to the TR_Lim_ VPD trait, will determine if this water conservation trait is suitable for deployment in cotton breeding programmes.

## Materials and Methods

Two glasshouse experiments were conducted in Narrabri, NSW during the 2019/2020 and 2020/2021 cotton seasons to assess the transpiration response of 10 different cotton genotypes to altered VPD conditions. Further details are described below.

### Genotype Selection

The germplasm used in this study is outlined in Table 1. Broadly, germplasm was selected on the basis of known yield performance, particularly under rainfed conditions, or previously published studies based on agronomic water use (Stiller et al., 2005) and/or the presence/absence of the limiting transpiration trait in response to atmospheric VPD (Devi and Reddy, 2018). Two genotypes, CS 50 and Siokra L23, in our study were identical to genotypes studied by Devi and Reddy (2018). Due to limited germplasm access as well as restrictions in Australia around growing Ingard® material that contains the single Cry1Ac protein, closely related material was selected as a substitute. Sicot 41 is closely related to FiberMax 9180 and DeltaPEARL is closely related to DP555 BG RR.

**Table 1:** Genotypes used in this study, including details on origin, release year and target environment.

Genotype	Origin	Release year	Target environment reason for inclusion
CSX2027	CSIRO, Narrabri AU	N/A	Rainfed
CSX8521	CSIRO, Narrabri AU	N/A	Rainfed; good, irrigated yield potential
CSX5422	CSIRO, Narrabri AU	N/A	Rainfed
Siokra L23	CSIRO, Narrabri AU	1993	Good agronomic WUE pair; no VPD breakpoint; examined by Devi and Reddy (2018)
CS 50	CSIRO, Narrabri AU	1992	Poor agronomic WUE pair; no VPD breakpoint; examined by Devi and Reddy (2018)
Sicot 41 (syn. FM958)	CSIRO, Narrabri AU	1999	VPD breakpoint; closely related to FM 9180 examined by Devi and Reddy (2018)
DeltaPEARL (Closely related to DP 555 BG RR)	Deltapine Australia Pty. Ltd., Goondiwindi AU	1999	VPD breakpoint; closely related to DP 555 BG RR examined by Devi and Reddy (2018)
RC-89(Syn. Surabhi)	Rasi Seeds, Attur India	1997	Irrigated and more recently rainfed in Tamil Nadu, India
Sicot 80BRF	CSIRO, Narrabri AU	2006	Rainfed
Sicot 746B3F	CSIRO, Narrabri AU	2016	Commercial irrigated Australian cultivar

### Growth Conditions

During each experiment, 100 plants (10 plants for each genotype) were grown at the University of Sydney’s Plant Breeding Institute at Narrabri (−30.271, 149.806) in temperature and humidity-controlled glasshouse facilities with natural light conditions (14 h d^−1^ of PPFD >2,000 mmol m^−2^ s^−1^). Eight seeds of each genotype were planted into 5 L pots filled with a 9:1 potting mix (Premium Potting Mix, Searles, Kilcoy QLD, Australia) and vermiculite mix (Debco Pty. Ltd., Tyabb Vic, Australia) on 9 December 2019 and 7 December 2020. To improve the nutrient status of the potting mix, 10 g of MULTIgro® (Incitec Pivot Fertilisers, Melbourne Australia) basal fertiliser was dissolved into the potting mix before planting. MULTIgro contains the nutrients N, P, K, S and Ca at 13.1%, 4.5%, 7.2%, 15.4% and 2.4%, respectively. Once seedlings had reached the three-leaf stage, pots were thinned to one plant per pot and a 1-cm layer of coarse perlite (Debco Pty. Ltd., Tyabb Vic, Australia) was placed on the surface of each pot. Prior to the initiation of VPD treatments, plants were grown at 32/18°C day/night. Plants were watered daily by hand to ensure non-limiting soil water conditions.

### VPD Treatments and Transpiration Calculations

When plants had reached first square [initiation of squares (floral buds); 35 days after planting (DAP)], two pots of each genotype were distributed across five separate glasshouse chambers and all pots were watered to field capacity and allowed to free drain overnight. The following morning, the surface of each pot was covered in aluminium foil to avoid soil surface evaporation during the measurement period.

Air temperature and relative humidity were adjusted to obtain five different VPD environments (target 2–6 kPa) across two consecutive days in each experiment (Supplementary Figure 1). The experimental design was a randomised complete block with five replicates. It was laid out as a 5 × 10 factorial design with five VPD levels (target 2–6 kPa) and 10 genotypes, with two pseudo-reps of each genotype in each VPD chamber. VPD treatments were replicated in time, where on the basis of a randomised design each chamber was exposed to each target VPD conditions once in each experiment, ensuring that the same VPD level was not assessed more than once in a given chamber or in more than one chamber at the same time. Once the chamber settings reached the desired VPD, the surface of pots were watered to field capacity, and the plants were allowed to acclimate to conditions for 1 h period before the initial pot weights were measured. Plants were then exposed to the set VPD for a further 1 h treatment period. Pots were reweighed to calculate the amount of water (g) used by the plant in the 1 h treatment period. Once plants in a chamber were exposed to a VPD treatment, the chamber settings were altered to generate the next target VPD level. This was done in a randomised complete block design, where each chamber was exposed to each target VPD treatment over a 2-day period (i.e., 36 and 37 DAP), with measurements occurring between 10 am and 5 pm. The measurements had to occur over 2 days to measure all VPD conditions in each chamber within daylight hours. Air temperature and relative humidity at canopy level inside each chamber were monitored a TinyTag data logger (model TGU 4017 Gemini Data Logger Ltd., West Sussex, United Kingdom). Despite all efforts to maintain temperature and humidity at the desired level, the facility’s ability to maintain temperature and humidity at the desired level was not always possible, particularly at high temperatures and high humidity.

Temperature and relative humidity across both experiments ranged from 29 to 48°C and 19%–75%, respectively. The range of VPD treatments imposed was between 1.4 and 7.2 kPa. The variation in VPD was primarily driven by altering temperature (Supplementary Figure 1).

Above ground biomass for each plant was harvested 38 DAP. Individual plant green leaf area was measured using the Li-COR LA-3100 leaf area meter (Li-COR Biosciences, Lincoln, NE, United States). The transpiration rate of each plant was calculated as the ratio of transpiration per leaf area and expressed as mg H_2_O m^−2^ s^−1^.

### Statistical Analysis

#### Transpiration Response to VPD of Different Cotton Genotypes

The presence of a breakpoint in a regression model of a given genotype’s transpiration vs. average VPD for the 1 h treatment period was assessed using the ‘segmented’ package (Muggeo, 2020) in R 4.0.1 (R Core Team, 2020). If the slopes were not significantly different (*p* < 0.05) using segmental linear regression, a simple linear regression was applied to all the data. Where genotypes expressed a two-segment response, the breakpoint regression analysis provides the VPD breakpoint (X_0_) between the two linear segments, as well as the slope of each segment and the standard error of each parameter of the regression model.

#### Modelling the Effect of VPD on Crop Water Use

Using the VPD-transpiration relationships developed in this study (Table 2), the daily transpiration of each genotype was calculated from sunrise to sunset (6 am to 8 pm; AEDT), expressed as g H_2_O m^−2^ day^−1^. VPD data were obtained for the Myall Vale weather station at the Australian Cotton Research Institute in Narrabri. Four days that represented the various VPD conditions throughout the peak flowering period were selected: extreme VPD day where maximum VPD = 6.0 kPa (17 January 2019); high VPD day where maximum VPD = 4.4 kPa (27 January 2021); moderate VPD day where maximum VPD = 3.2 kPa (21 January 2021); and low VPD day where maximum VPD =1.0 kPa (6 February 2021; Table 3). These VPD conditions reflected actual environmental conditions in the field, demonstrating the VPD extremes encountered during peak flowering. Genstat version 19 was used to perform a two-sample *t*-test to analyse daily water use of TR_Linear_ and TR_Lim_ transpiration response at each VPD environment. Significance was determined using 5% level of probability.

**Table 2:** Breakpoint, slope, and *r*^2^ results from regression analyses of the transpiration response of 10 cotton genotypes to VPD.

Genotype	Breakpoint	Slope 1	Slope 2	*r* ^2^
	(X_0_) ± SE			
Sicot 41	5.3 ± 0.5	13.51	0.01	0.54
CSX5422	4.3 ± 0.5	16.91	2.51	0.50
Sicot 80BRF	4.8 ± 0.5	14.45	0.45	0.45
Sicot 746B3F	6.6 ± 2.3	12.17	−38.72	0.50
DeltaPEARL	4.5 ± 0.4	17.39	2.54	0.57
CSX2027	Linear	11.87		0.48
CS 50	Linear	12.39		0.58
RC-89	Linear	9.64		0.44
Siokra L23	Linear	12.77		0.60
CSX8521	Linear	10.48		0.42

**Table 3:** Environmental conditions of 4 days with representative VPD signals.

Environmental conditions	VPD environment			
	Extreme VPD	High VPD	Moderate VPD	Low VPD
T_min_ (°C)	26.8	25.1	18.6	21.3
T_max_ (°C)	40.7	37.7	32.8	25.0
RH_min_ (%)	22.0	31.0	35.0	67.0
RH_max_ (%)	53.0	60.0	88.0	93.0
VPD_min_ (kPa)	1.9	1.3	0.3	0.2
VPD_max_ (kPa)	6.0	4.4	3.2	1.0

*Data were collected from Narrabri, Australia, during the peak flowering of cotton production*.

#### Effects of Water and VPD Environments During Peak Flowering on Cotton Lint Yield

Yield data from historic dryland and managed stress environment (MSE) experiments (Conaty et al., 2018) conducted as part of the CSIRO cotton breeding programme at Bellata, Darling Downs, and Narrabri from 2006 to 2020 were obtained for four genotypes: CSX2027 (*n* = 36), CSX8521 (*n* = 35), Sicot 80BRF (*n* = 44), and Sicot 746B3F (*n* = 44), where *n* = the number of data points available. Each of these cultivars were selected because yield data was available across several years and locations. Weather data were used to calculate total rainfall and the mean maximum VPD during the peak flowering period in each year. Peak flowering was defined as between 1,000- and 1,450-day degrees. This study was limited to the peak flowering window because although an indeterminate plant, cotton is most sensitive to water stress during the peak flowering window (Grimes, 1970; Hearn and Constable, 1984). Genstat version 19 was used to fit a Generalised Linear Model to determine the effects of water and VPD environments at peak flowering on final lint yield. To test the model effects, data were analysed by a successive forward stepwise regression. A generalised linear regression analysis was conducted, where the response variate was fitted to a model based on the remaining parameters of interest as well as all significant interactions between these parameters. The regression analysis was then used to calculate the relationship between yield and water availability during peak flower at a given VPD for two of the four genotypes: CSX2027 and Sicot 80BRF. The study was limited to these two genotypes as the dataset for the other two genotypes contained limited yield data across seasons with differing VPD and availability of water during peak flowering. However, the generalised linear model analysed four genotypes (two TR_Linear_ and two TR_Lim_).

## Results

### Transpiration Response to VPD of Different Cotton Genotypes

**Figure 1:**
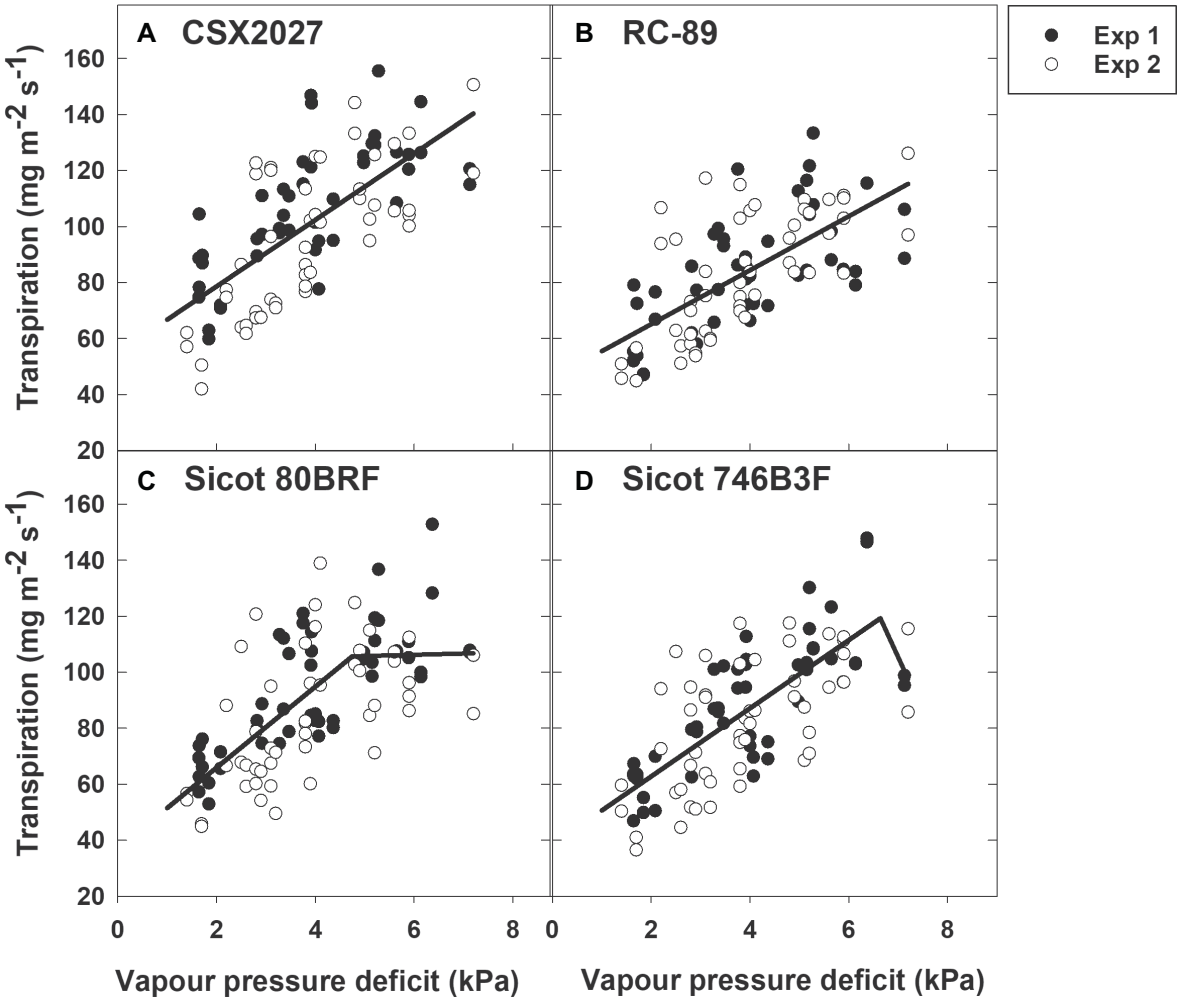
Transpiration response of four cotton genotypes to vapour pressure deficit (VPD); **(A)** CSX2027, **(B)** RC-89, **(C)** Sicot 80BRF, and **(D)** Sicot 746B3F. Data from experiment 1 (Exp 1) is shown by black symbols and experiment 2 (Exp 2) shown by white symbols. Regression lines are shown by the black solid lines.

The 10 genotypes screened displayed a range of transpiration responses to VPD (Table 2; Figure 1; Supplementary Figure 2). Half of the genotypes were characterised by the two-segmental analysis with a break point (X_O_; considered to be TR_Lim_), whilst the remaining genotypes exhibited a linear increase in transpiration in response to VPD whilst under well-watered conditions. Genotypes that were identified as TR_Linear_ included CSX2027, CS 50, RC-89, Siokra L23 and CSX8521. Genotypes that were identified as expressing the TR_Lim_ trait included Sicot 41, CSX5422, Sicot 80BRF, Sicot 746B3F and DeltaPEARL. The *r*^2^ for the two-segmented regressions ranged from 0.45 to 0.57, with the breakpoints ranging from 4.3 ± 0.5 to 6.6 ± 2.3 kPa. The secondary slope of these regressions ranged from −38.72 to 2.54 mg H_2_O m^−2^ s^−1^. The initial slope of the genotypes with a two-segmented response ranged from 12.17 to 17.39 mg H_2_O m^−2^ s^−1^. These slopes were greater than those genotypes that did not express a two-segmented response in transpiration to VPD (*p* = 0.017), where slopes ranged from 9.64 to 12.77 mg H_2_O m^−2^ s^−1^ and *r*^2^ of the linear regressions ranging from 0.42 to 0.60.

### Modelled Water Use of Cotton Genotypes at Different VPD Environments

**Figure 2:**
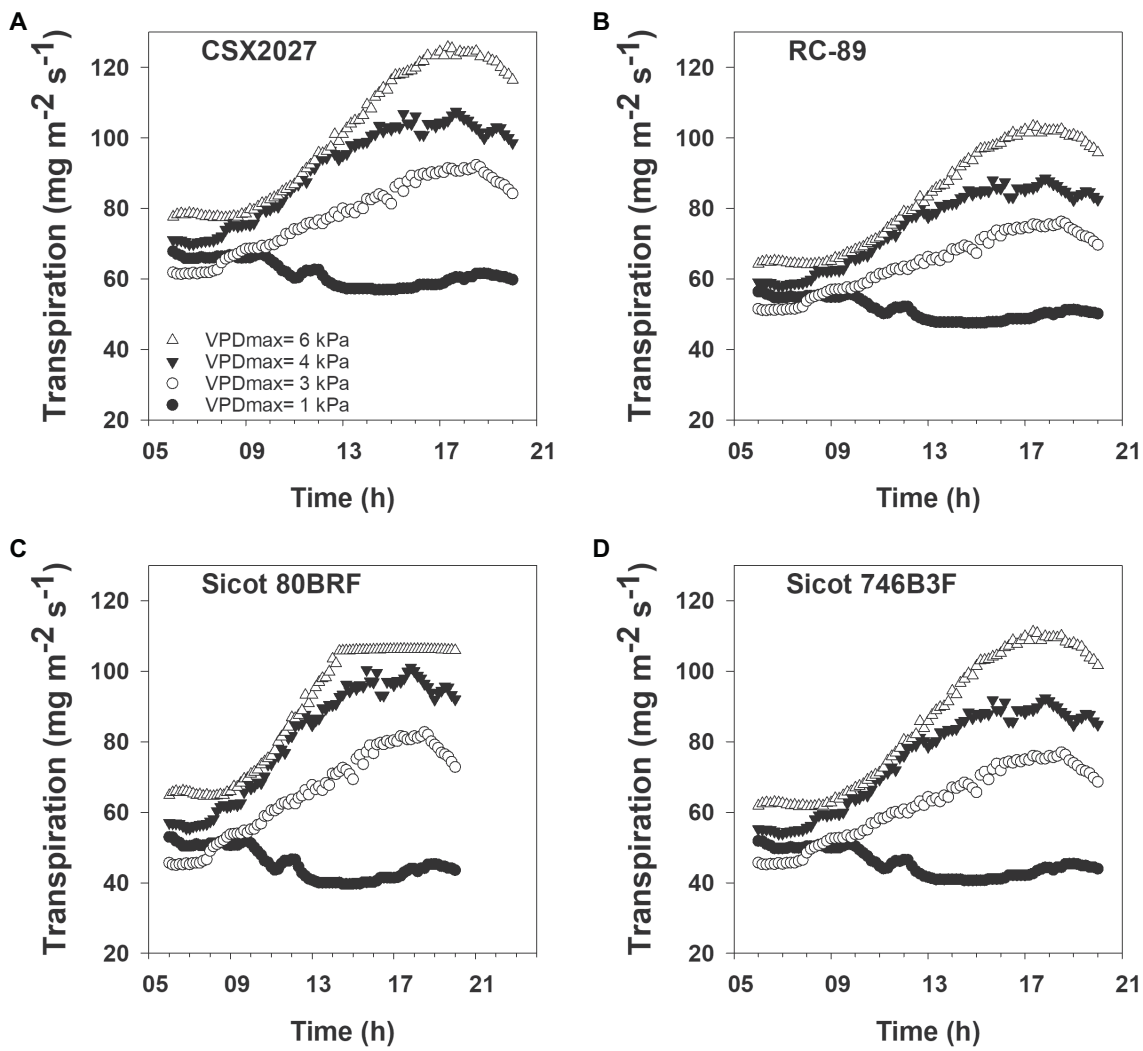
Four cotton genotypes **(A)** CSX2027, **(B)** RC-89, **(C)** Sicot 80BRF, and **(D)** Sicot 746B3F, with differing transpiration responses to representative VPD conditions in Narrabri NSW from sunrise to sunset (6 am to 8 pm; AEDT).

Genotypic variability in the transpiration response affected the way water was used throughout the day. Despite having a linear transpiration response (TR_Linear_), RC-89 had a lower slope (9.6) that resulted in lower transpiration rates in high VPD environments, with a transpiration rate of up to 103 mg m^−2^ s^−1^ during a 6 kPa day (Figure 2). In contrast, of the varieties that displayed a TR_Linear_ response, CSX2027 had a steeper slope that resulted in higher transpiration rates of up to 126 mg m^−2^ s^−1^ during a 6 kPa day. Transpiration responses of TR_Lim_ genotypes depended on the breakpoints as to the shape of the response curve. Genotypes that had a lower VPD breakpoint had flatter plateau in their transpiration rate where transpiration was limited during high VPD environments (e.g., Sicot 80BRF) compared with genotypes that had higher VPD breakpoints (e.g., Sicot 746B3F). For example, Sicot 80BRF demonstrated a distinct plateau with a maximum transpiration rate of 106 mg m^−2^ s^−1^ during the 6 kPa day. Despite our data showing that Sicot 746B3F has a VPD breakpoint of 6.64 kPa, a TR_Lim_ response would not have been initiated in the environmental conditions observed in our desktop study, resulting in a maximum transpiration rate of 111 mg m^−2^ s^−1^ during the 6 kPa day.

**Figure 3:**
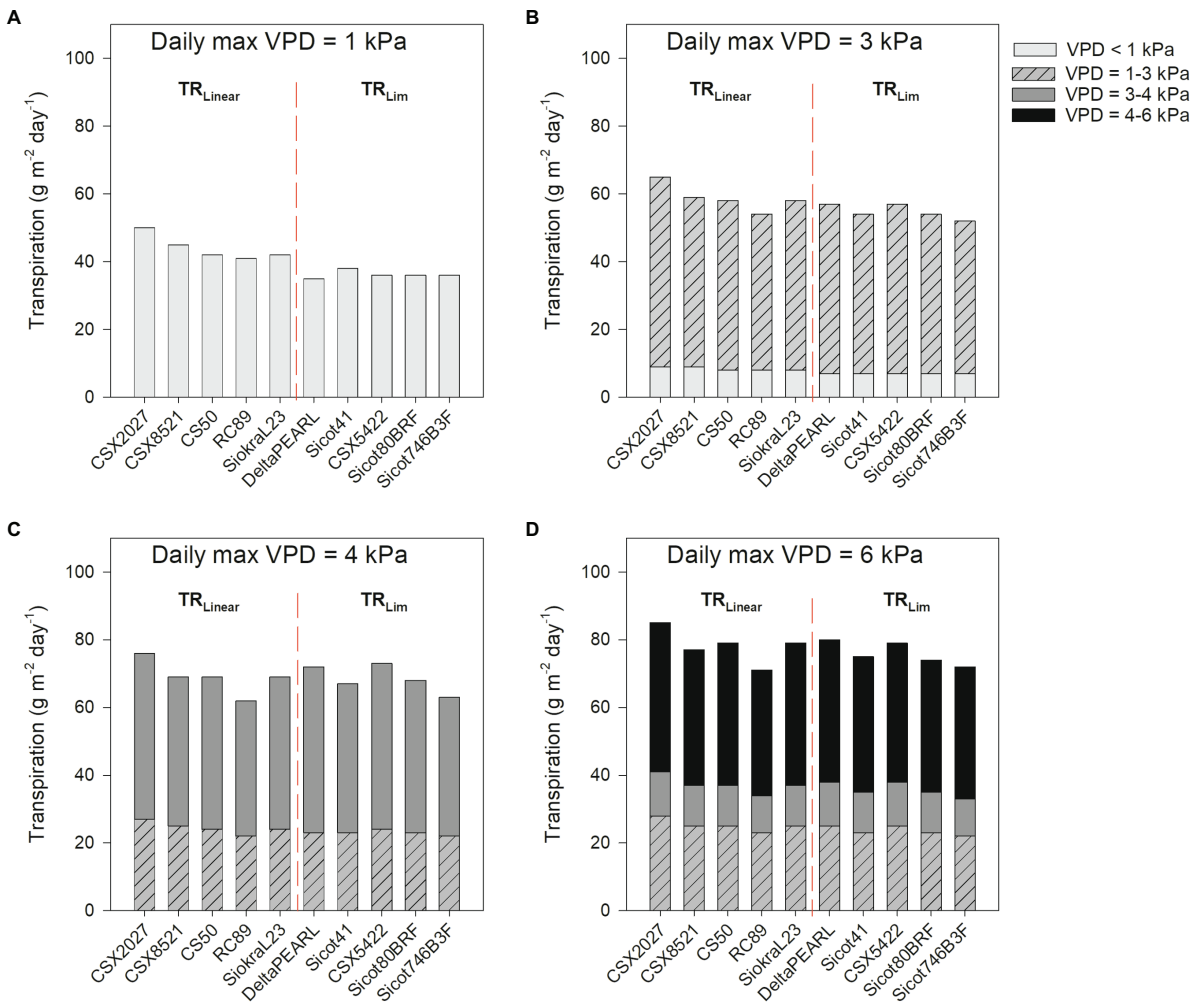
Daily transpiration (g m^−2^ day^−1^, from sunrise until sunset) of 10 cotton genotypes for 4 days representing differing VPD conditions in Narrabri, Australia, during peak flowering. **(A)** Max VPD = 1.0 kPa (6 February 2021), **(B)** Max VPD = 3.2 kPa (21 January 2021), **(C)** Max VPD = 4.4 kPa (27 January 2021), and **(D)** Max VPD = 6.0 kPa (17 January 2019). VPD environments range from VPD < 1 kPa (light grey), 1–3 kPa (hashed grey), 3–4 kPa (dark grey), and 4–6 kPa (black). The red dashed line separates TR_Linear_ and TR_Lim_ genotypes.

Averaged across all five genotypes within each transpiration response trait, TR_Lim_ had 17% lower daily transpiration than TR_Linear_ in low VPD environments (VPD =1.0 kPa; *p* = 0.007), but there were no significant differences in daily transpiration in high VPD environments (VPD = 4.4 kPa and VPD = 6.0 kPa; *p* > 0.05; Figure 3; Table 4). Although statistical differences were not observed, the TR_Linear_ genotype CSX2027 consistently had the greatest daily transpiration rate in each VPD environment. The TR_Lim_ genotypes, DeltaPEARL had the lowest daily transpiration at low VPD (35 g m^−2^ day^−1^, VPD = 1.0 kPa), but amongst the highest transpiration rates in high VPD environments (72 and 80 g m^−2^ day^−1^, VPD = 4.4 kPa and VPD = 6.0 kPa, respectively), despite a transpiration breakpoint at 4.52 kPa. In comparison, TR_Lim_ genotype Sicot 746B3F is amongst the lowest daily transpiration at each VPD environment, despite a transpiration breakpoint at 6.64 kPa.

**Table 4:** Two-sample *t*-test results for daily water use of cotton with differing transpiration response at each VPD environment.

Daily VPD environment	TR_Linear_ water use (g m^−2^ day^−1^)	TR_Lim_ water use (g m^−2^ day^−1^)	*t*, *df*	*p*
VPD_Max_ = 1.0 kPa	43.97	36.33	4.70, 4.57^*^	**0.007**
VPD_Max_ = 3.2 kPa	58.71	55.02	1.80, 8	0.109
VPD_Max_ = 4.4 kPa	69.12	68.60	0.19, 8	0.857
VPD_Max_ = 6.0 kPa	77.91	76.40	0.54, 8	0.606

*Values in bold are significant at *p* < 0.05*.

* *Evidence of unequal variances*.

### Effects of Water and VPD Environments During Peak Flowering on Lint Yield

The generalised linear model used to assess the effects on lint yield of four cotton genotypes indicated that available water during peak flowering accounted for 27.7% of the variation (Table 5). The cumulative addition of mean VPD_Max_ during peak flowering (+13.0%), water × VPD_Max_ (+24.3%), and VPD_Max_ × genotype (+1.9%) accounted for a total of 66.7% of the variation in lint yield. Therefore, the best fitting model for predicting lint yield was Water + VPD_Max_ + Water × VPD_Max_ + VPD_Max_ × Genotype.

**Table 5:** Effect of water and VPD environment during peak flowering on lint yield of four cotton genotypes (CSX2027, CSX8521, Sicot 80BRF, and Sicot 746B3F) grown across Australian cotton regions from 2006 to 2020.

Explanatory variable	% Variation	Change
Water	27.7	-
VPD_Max_	+13.0	0.001
Genotype	0	0.215
Water × VPD_Max_	+24.3	0.001
Water × Genotype	0	0.200
VPD_Max_ × Genotype	+1.9	0.009
Water × VPD_Max_ × Genotype	0	0.521
Total variation	66.7	-

*Variation (%) accounted for by including each term in the model is shown. Change was deemed significant at *p* < 0.05. Peak flower is defined as between 1,000- and 1,450-day degrees*.

**Figure 4:**
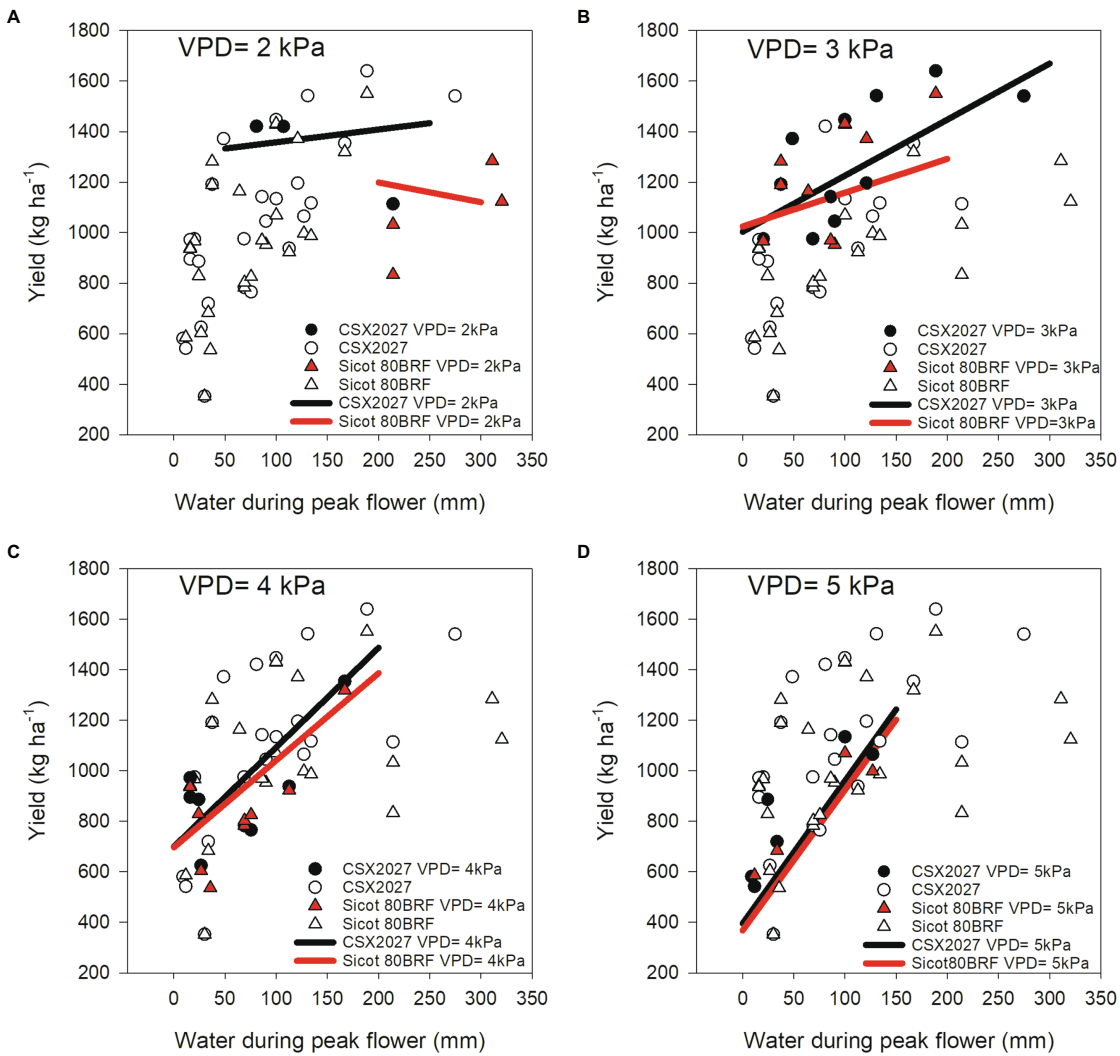
The observed relationship between water during peak flower (mm) and yield (kg ha^−1^) for CSX2027 and Sicot 80BRF as influenced by atmospheric VPD. CSX2027 is represented by circles and Sicot 80BRF is represented by triangles. Shaded data points (black = CSX2027, red = Sicot 80BRF) represent the relevant VPD in each panel; **(A)** VPD = 2 kPa, **(B)** VPD = 3 kPa, **(C)** VPD = 4 kPa, and **(D)** VPD = 5 kPa. Regression lines for each VPD are shown (black = CSX2027, red = Sicot 80BRF).

The relationship between atmospheric (VPD) and soil (water availability during peak flower) environments and their association with cotton yield of two genotypes with differing TR_Lim_ VPD traits (CSX2027 = TR_Linear_ and Sicot 80BRF = TR_Lim_) are shown in Figure 4. When there is more water available during peak flowering and at lower VPD environments (i.e., VPD = 2 kPa), CSX2027 had greater yields than Sicot 80BRF. As suggested by the intercept of the regression lines, at moderate to high VPD (i.e., VPD = 3 kPa and VPD = 4 kPa) the two cultivars had equivalent yields at low water availability, but the TR_Linear_ variety’s yield (CSX2027) was higher when water availability was increased across all VPD ranges, with exception of where VPD = 5 kPa. At extremely high VPD (i.e., VPD = 5 kPa), yield response to available water during peak flower was very similar in both CSX2027 and Sicot 80BRF.

## Discussion

### Cotton Genotypes Have Differing Transpiration Responses to VPD Environments

Our study showed variation amongst 10 cotton genotypes assessed for their transpiration response to higher VPDs, thereby supporting our first hypothesis that there is genetic variability to the TR_Lim_ VPD trait in germplasm present in the Australian cotton breeding programme. Five of the genotypes that we studied limited their transpiration rate in high VPD environments (between 4.3 kPa and 6.6 kPa) thereby possessing a TR_Lim_ VPD trait whereas the other five genotypes continued to increase their transpiration rate in high VPD environments, depicting a TR_Linear_ VPD response. The observed breakpoint response was likely caused by stomatal closure to prevent water loss under high evaporative demand. Similarly, Devi and Reddy (2018) also found genotypic differences in cotton transpiration response to VPD, with some genotypes also limiting transpiration in high VPD environments. Common genotypes between the two studies included CS 50 and Siokra L23, with DeltaPEARL and Sicot 41 included in our study as closely related germplasm to DP555 BG RR and FiberMax 9180, respectively (Table 1). Our data closely aligned with Devi and Reddy (2018), whereby both CS 50 and Siokra L23 did not limit transpiration at high VPD. In comparison, DeltaPEARL and DP555 BG RR, and Sicot 41 and FiberMax 9180 all demonstrated a limiting transpiration rate. A consistent and stable transpiration response amongst these genotypes in these two studies suggests potential for incorporation of these transpiration limiting traits in a breeding programme to improve germplasm better suited to high VPD environments.

### How Do Environmental Conditions Affect the TR_Lim_ VPD Trait?

All VPD breakpoints in our study occurred between 4.3 and 6.6 kPa compared with a VPD breakpoint around 2 kPa reported by Devi and Reddy (2018). The VPD environment during our study ranged from 1.4 to 7.2 kPa compared with a much smaller range from 0.9 to 3.3 kPa in the study by Devi and Reddy (2018). The difference in the VPD breakpoints may be the result of environmental conditions and plant adaptation, which indicates that the expression of the trait may interact with the testing environment. For example, Devi and Reddy (2018) conducted their experiments in growth chambers under lower light conditions (16 h d^−1^ photosynthetic photon flux density (PPFD) of 800 mmol m^−2^ s^−1^) whilst our experiments were conducted under natural summer light conditions (14 h d^−1^ of PPFD >2000 mmol m^−2^ s^−1^). As cotton is highly responsive to light environment, it is possible that the low light experimental conditions used in the Devi and Reddy (2018) experiments may have lowered the observed TR_Lim_ VPD breakpoints. However, VPD breakpoints for other species are also typically lower than what we observed in our study. For example, transpiration control occurred between 1.39 and 2.50 kPa in *Agrostis stolonifera* (Shekoofa et al., 2016a) in a study that demonstrated limited transpiration responses in high VPD environments can also be induced chemically. This could be partially due to the lower VPD environments that many plant species grow, and are therefore, tested in.

High VPD most commonly occurs in high temperature environments (Supplementary Figure 1), and therefore it is important to understand if the TR_Lim_ VPD trait in cotton withstands high temperatures. Recent research has identified some dynamic expression and adaptation of the TR_Lim_ VPD trait with respect to thermal and temporal environments, particularly under high temperatures (Shekoofa et al., 2021). In a chamber study, Shekoofa et al. (2016b) showed that five maize hybrids that expressed the TR_Lim_ trait at 32°C did not express the TR_Lim_ responses at 38°C. This has also been shown in cotton, where some genotypes lost the TR_Lim_ trait at 38°C (Shekoofa et al., 2021). The loss of the VPD breakpoint at higher temperatures indicates an increase in hydraulic conductance at high temperatures (Choudhary et al., 2014). High temperature and VPD responses in plants are complex, with stomata constantly adjusting to changes in VPD, leaf water potential, and leaf temperature to control transpiration rates. Devi and Reddy (2018) showed variation amongst 17 cotton genotypes tested for their transpiration response to increasing VPD under 32°C. In contrast, the maximum temperatures in our study reached 48°C, enabling us to achieve a broader range of VPD environments that has already been seen in field conditions, and may continue to be seen in the future. Whilst a greater range of VPDs were achieved by taking this approach, it must be acknowledged that considering the above studies conducted by Shekoofa et al. (2016b, 2021) the associations observed between VPD and transpiration in our study may be influenced by the altering of temperature when generating VPDs. However, we believe that this approach better reflects the likely environment experienced in cotton production where increases in VPD are largely driven by changes in both temperature and humidity, not just one parameter. However, it is important to note that both physical and biochemical limitations are also likely to occur under extremely high temperature conditions. Further research is required to understand if these, or other, factors alter the expression of the TR_Lim_ VPD water conservation trait in the genotypes of cotton in our study, and thus whether the heritability of the TR_Lim_ VPD trait will enable it to be incorporated into a breeding programme.

The environment in which the germplasm originated, and the selection pressures of this environment, may have influenced the presence and the degree of expression of the TR_Lim_ VPD trait in germplasm resources. Although bred in Australia, the pedigree of DeltaPEARL was strongly influenced by germplasm from Scott, Mississippi, United States (Delta and Pine Land Co.). Specifically, DeltaPEARL was developed from a controlled cross of DP 5816, which was bred in Mississippi, and Sicala 34, which was bred in Australia. Scott, MS is characterised by a humid subtropical climate with maximum daily temperatures averaging 33.6°C and rainfall averaging 96 mm in the month of July, when peak flowering would be expected in this region. This breeding environment may have influenced the lower VPD breakpoint observed in the DeltaPEARL genotype in our study. In contrast, varieties such as Sicot 41, Sicot 80BRF and Sicot 746B3F have been bred in Australia and tend to better suit Australian environments which are typically hotter and drier (Liu et al., 2013), potentially influencing the slightly higher VPD breakpoints seen in our study. Therefore, it is important to test responses to a broad range of VPD environments that are likely to occur in a production system.

### Does the TR_Lim_ VPD Trait Conserve Water?

As well as understanding if there are VPD breakpoints and where they may occur, we also investigated how the transpiration responses differed. The initial slope of the genotypes with a two-segmented response ranged from 12.17 to 17.39 mg H_2_O m^−2^ s^−1^. These slopes were greater than those genotypes that did not express a two-segmented response of transpiration to VPD (*p* = 0.017), where slopes ranged from 9.64 to 12.77 mg H_2_O m^−2^ s^−1^. Thus, although genotypes may limit their transpiration in high VPD environments, our data shows that TR_Lim_ cotton can transpire significantly more water at lower VPD conditions. This may be an important factor when selecting genotypes suited to limited soil water environments and may be investigated in future studies, and specifically with consideration to field environments.

Temperature and humidity, and thus VPD, changes both throughout the day as shown in Figure 2, and throughout the growing season. Therefore, plants are constantly responding to changing VPD environments. Additionally, crops grown throughout different production areas are likely to experience different environmental conditions (Gonias et al., 2012), which is important to consider when expanding an industry into new regions. A key theory of TR_Lim_ behaviour is that at midday under high VPD conditions, there is conservation of soil water, which would be particularly beneficial in rainfed and partially irrigated cotton systems. However, our modelled data (Figures 2, 3; Table 4) suggests that although genotypes with the TR_Lim_ VPD trait limit their water use in high VPD environments, a greater initial rate of transpiration in lower VPD environments (i.e., slope 1 ranging from 12.17–17.39 for TR_Lim_ genotypes compared with slope 1 ranging from 9.64–12.77 for TR_Linear_ genotypes; Table 2) could negate the potential water conservation in high VPD environments, thereby disproving our second hypothesis that genotypes with a TR_Lim_ VPD trait use less water in high VPD environments. Thus, higher rates of transpiration earlier in the day could negate water conservation in high VPD environments. Alternatively, higher rates of transpiration earlier in the day could ensure greater productivity during a period of lower atmospheric demand for water resources by the plant, subsequently making them more agronomically water use efficient. However, this should be confirmed with additional field studies as rooting dynamics could also be an important factor determining varieties suited to water limited production.

### How VPD Environments and Water Availability During Peak Flower Affect Yield of Different Cotton Genotypes

In a modelled study, Sinclair et al. (2005) found a 9–13% improvement in Australian sorghum production when transpiration rate was limited in high VPD environments. This was attributed to higher yields predominately in dry, low-yielding years in which growers were typically more economically vulnerable. However, this assumes that the transpiration rate of TR_Lim_ genotypes was not significantly greater than TR_Linear_ genotypes in low VPD environments, as seen in our study. Thus, it is important to consider what effects a limited water trait and potential differences in water use have on yield.

Our data showed that available water during peak flowering was the primary driver of yield, accounting for 27.7% of the variation in cotton yield (Table 5). Sequentially adding mean VPD_Max_ during peak flowering accounted for a further 13.0% of the variation. Further including the interactive terms Water × VPD_Max_ and VPD_Max_ × Genotype significantly improved the cotton yield model, accounting for 66.7% of the variation in cotton yield. Therefore, genotype interactions with the VPD environment were small, but significant predictors of yield, supporting our third hypothesis that variation in yield responses of cotton genotypes is linked with the VPD environment and water availability during peak flowering period.

The effects of VPD environments and available water during peak flower on the yield of genotypes with differing TR_Lim_ VPD traits is shown by our comparison of two genotypes, CSX2027 and Sicot 80BRF (Figure 4). Our data show that the yield of the TR_Linear_ genotype (CSX2027) was much greater than the yield of the TR_Lim_ genotype (Sicot 80BRF) at lower VPD environments and especially when there is more available water during peak flower. Additionally, CSX2027 also had more stable yield across water availability under low VPD environments than Sicot 80BRF. However, in higher VPD environments (VPD > 3 kPa), the yield of Sicot 80BRF was comparable to yield for CSX2027, particularly when there was low water availability during peak flower. Our data also suggests that ultimately variety performance, either with or without the limiting transpiration trait, is limited by the yield potential of the variety. CSX2027 has a higher yield potential than Sicot 80BRF, simply because it was developed 6 years later from parents with improved performance characteristics. It is likely that these differences in yield potential would have altered the outcome of the regression model. Additionally, whilst this regression model was developed from an extensive field experimental data set encompassing multiple seasons (*n* = 18) and testing sites (*n* = 7), this study is limited by the number genotypes used in this study, and the genotypes differing genetic backgrounds. Although it is likely that studies using Near Isogenic Lines (NILs) are not possible, as the TR_Lim_ VPD phenotype is a complex (polygenic) trait, this must be acknowledged when interpreting the observed relationship between yield performance and VPD environment with respect to TR_Lim_ VPD trait presence/absence. For growers to adopt cultivars that exhibit the TR_Lim_ VPD trait, the germplasm must have a yield potential that enables it to take advantage of in-crop rainfall events. For example, although transpiration rates were lower in RC-89, yield potential is also lower than the other genotypes studied, are therefore RC-89 is a less desirable than other higher-performing cultivars. Therefore, further studies need to determine if the TR_Lim_ VPD trait is inherently associated with specific performance limitations, or if breeding and selection can exploit this trait in germplasm with high yield potential. In addition, it is also necessary to consider the implications of overall morphology on water use and ultimately water use efficiency. Even though a genotype may have a greater rate of transpiration at the leaf-level, if it has a smaller habit or reduced leaf area, overall crop-level water use may be less compared with other genotypes, which was not accounted for in our analysis at the field scale.

It is important to note that our glasshouse study investigated the transpiration responses of well-watered cotton plants. Therefore, future research should test transpiration responses under water-limited conditions. Additionally, transpiration responses to interactive atmospheric and soil water deficits have not been explored in Australian germplasm and should be the focus of future research. This is important as water limited production traits are the target breeding environment for these water conservation traits. Lobell et al. (2014) found that despite cultivar improvements in maize, the sensitivity of maize yields to soil water deficits associated with higher VPD has increased. However, that is likely due to increased sowing density rather than genotypic factors. Although sowing density is managed to maximise the amount of water available to the crops, particularly in rainfed cotton systems, there may be genetic resilience in Australian germplasm to withstand combined atmospheric and soil water deficits that could be explored.

Finally, although these studies provide important insight into TR_Lim_ VPD traits, we must acknowledge that these experiments cannot be directly used for breeding because the experimental complexity does not allow for breeding and selection beyond the identification of parents for crossing. Thus, after determining the value of the trait, secondary or associated traits will need to be identified to enable selection of breeding material expressing the TR_Lim_ VPD trait, and further integration into a breeding programme.

## Conclusion

This study identified genetic variation to the limiting transpiration VPD trait within cotton genotypes in the CSIRO cotton breeding programme. Five genotypes were identified as expressing the TR_Lim_ VPD trait, where transpiration was limited from 4.3 to 6.6 kPa, depending on genotype. However, our modelling study indicates that the TR_Lim_ VPD trait may not necessarily reduce overall plant water use due to greater transpiration rates in lower VPD environments negating the water conservation in high VPD environments. Although this study demonstrated that G × E × M variables accounted for the 66.7% of the variation in cotton yield, yield performance between transpiration responses in high VPD environments were comparable. Yield performance of the TR_Lim_ genotype was improved in some high VPD environments but is unlikely to out-perform a genotype with lower yield potential. Therefore, although it may be possible for a TR_Lim_ VPD trait to improve cotton yield in projected future hotter, drier climatic conditions, overall crop water requirements may be the same. These findings may have important implications for the use of this trait in breeding programmes. As G × E × M interactions are associated with this trait, these concepts should be assessed in the field with greater datasets. Importantly, these studies will ascertain the potential value of the trait to cotton breeding as well as its heritability. Improved understanding of integrated plant- and crop-level genotypic responses to VPD environments, particularly under interactive atmospheric and soil water deficits, will enhance our understanding of germplasm responses to water deficits. This knowledge will benefit cotton breeding and production in both rainfed and semi-irrigated cotton systems, thereby meeting the agricultural challenges of the twenty-first Century.

## Data Availability Statement

The datasets presented in this article are not readily available because all data are governed by IP restrictions applicable to Cotton Breeding Australia projects. Requests to access the datasets should be directed to warwick.stiller@csiro.au.

## Author Contributions

WC conceived the study and reviewed the manuscript. KB and WC contributed to the collection and analysis of the data. KB wrote the manuscript. All authors contributed to the article and approved the submitted version.

## Funding

This research was funded by the Cotton Breeding Australia (CBA24), a joint venture between the CSIRO and Cotton Seed Distributors Ltd.

## Conflict of Interest

The authors declare that the research was conducted in the absence of any commercial or financial relationships that could be construed as a potential conflict of interest.

## Publisher’s Note

All claims expressed in this article are solely those of the authors and do not necessarily represent those of their affiliated organizations, or those of the publisher, the editors and the reviewers. Any product that may be evaluated in this article, or claim that may be made by its manufacturer, is not guaranteed or endorsed by the publisher.
