# Supplementary material for: Understanding and Exploiting Transpiration Response to Vapor Pressure Deficit for Water Limited Environments
Source: Front Plant Sci. 2022 May 10;13:893994. doi: 10.3389/fpls.2022.893994 (PMC9127727; doi:10.3389/fpls.2022.893994)
Supplement: Supplementary file 1 [file Data_Sheet_1.docx]

Supplementary Material


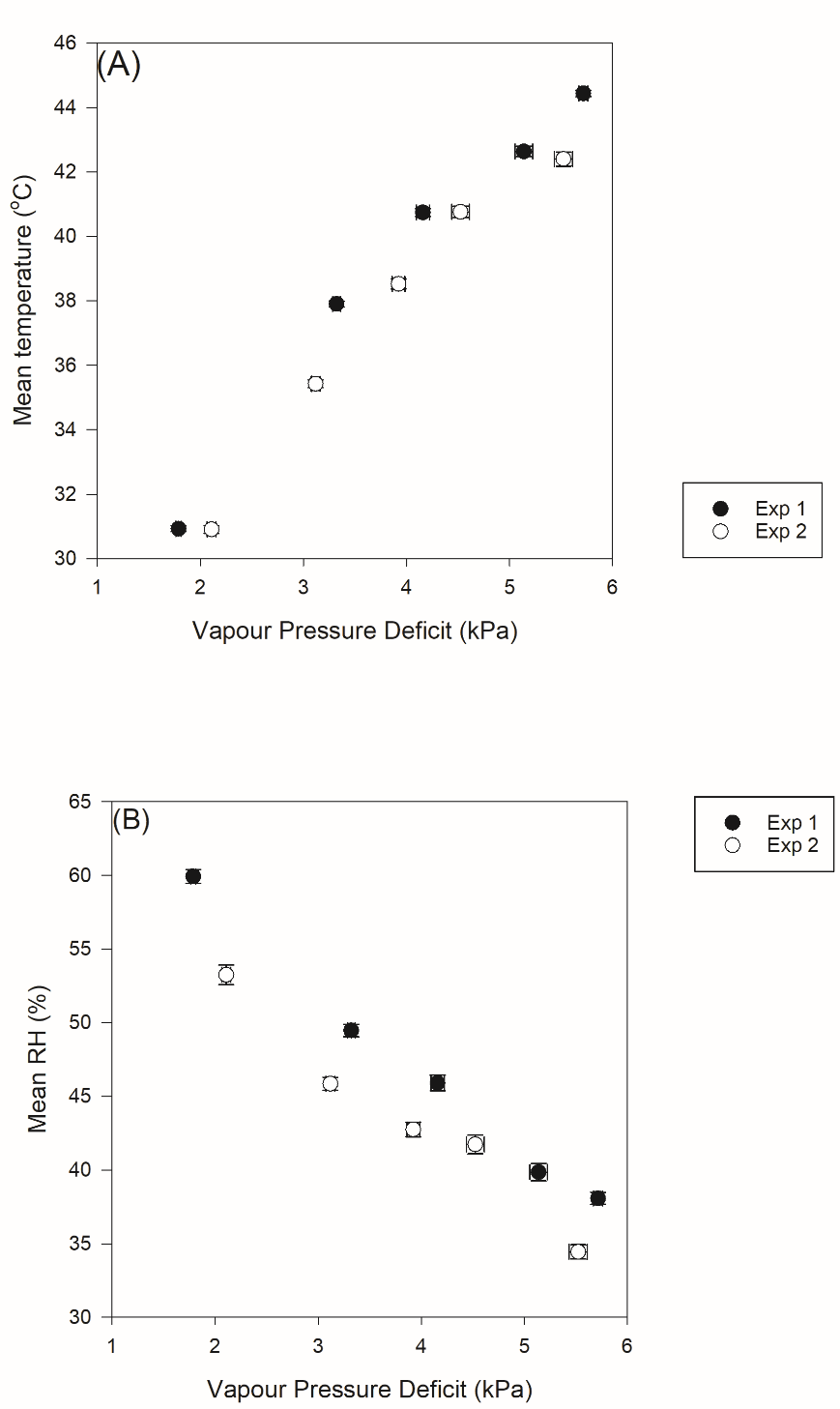


Supplementary Figure 1: (A) Mean temperature and (B) relative humidity (RH) in each vapour pressure deficit (VPD; kPa) environment in the glasshouse. Values represent the mean of five chambers in experiment 1 (black symbols) and experiment 2 (white symbols) ± standard error.


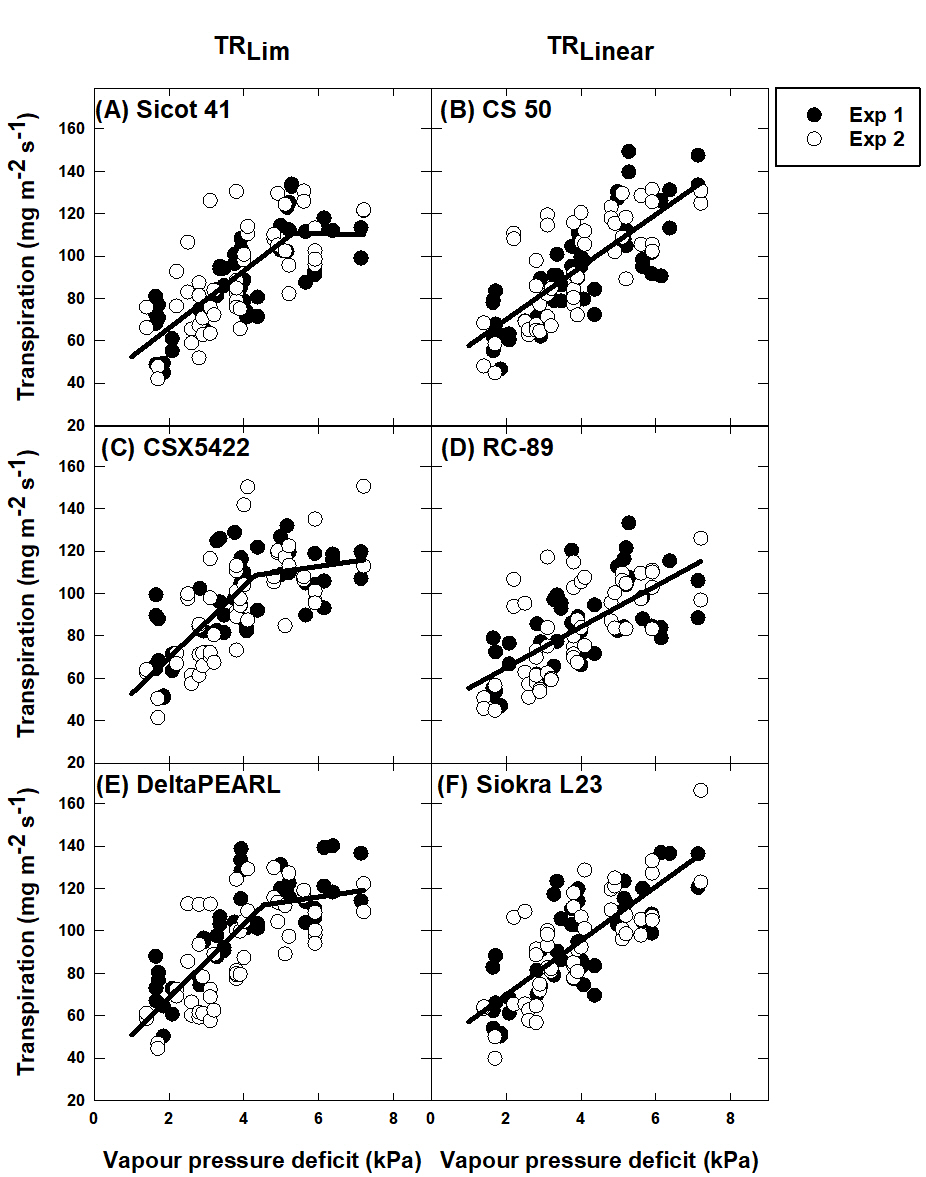


**Supplementary Figure 2: Transpiration response of six cotton genotypes to vapour pressure deficit (VPD); (A) Sicot 41, (B) CS 50, (C) CSX5422, (D) RC-89, (E) DeltaPEARL and (F) Siokra L2. TRLim genotypes were identified as Sicot 41, CSX5422 and DeltaPEARL (A, C, and E, respectively) and TRLinear genotypes were identified as CS 50, RC-89 and Siokra L23 (B, D and F, respectively). Data from experiment 1 (Exp 1) is shown by black symbols and experiment 2 (Exp 2) shown by white symbols. Regression lines are shown by the black solid lines.**
